# The Mitochondrial DNA Northeast Asia CZD Haplogroup Is Associated with Good Disease-Free Survival among Male Oral Squamous Cell Carcinoma Patients

**DOI:** 10.1371/journal.pone.0049684

**Published:** 2012-11-21

**Authors:** Chih-Hsiung Lai, Shiang-Fu Huang, I-How Chen, Chun-Ta Liao, Hung-Ming Wang, Ling-Ling Hsieh

**Affiliations:** 1 Graduate Institute of Biomedical Sciences, Chang Gung University, Tao-Yuan, Taiwan; 2 Department of Otolaryngology, Head and Neck Surgery, Chang Gung Memorial Hospital, Tao-Yuan, Taiwan; 3 Division of Hematology/Oncology, Department of Internal Medicine, Chang Gung Memorial Hospital, Tao-Yuan, Taiwan; 4 Department of Public Health, Chang Gung University, Tao-Yuan, Taiwan; Institute of Molecular and Cell Biology, Singapore

## Abstract

Reprogramming of energy metabolism in cancer cells has been directly/indirectly linked to mitochondria and mitochondrial functional defects and these changes seem to contribute to the development and progression of cancer. Studies have indicated that mitochondrial DNA haplogroups are associated with risk in relation to various diseases including cancer. However, few studies have examined the effect of haplogroups on cancer prognosis outcome. In order to explore the role of haplogroups on oral squamous cell carcinoma (OSCC) prognosis, the mitochondrial genomes of 300 male OSCC patients were comprehensively analyzed by direct sequencing. They were then haplotyped and grouped into four major geographic haplogroups, namely the East Asia AN, Southeast Asia RBF, East Asia MGE and Northeast Asia CZD groups. The Kaplan-Meier plot analysis indicated that individuals who were members of the CZD haplogroup showed a significant association with better disease-free survival (DFS) than the other three haplogroups and this phenomenon still existed after adjusting for tumor stage, differentiation and age at diagnosis (hazard ratio = 0.55; 95% CI = 0.36–0.84). In addition, an interaction between membership of the RBF haplogroup and radiotherapy/chemo-radiotherapy in DFS was also identified. The results strongly support the hypothesis that an individual’s haplogroup, by defining their genomic background, plays an important role in tumor behavior and mitochondrially-targeted anticancer drugs are promising future therapeutic approaches.

## Introduction

Oral cancer is amongst the most prevalent cancers worldwide and incidence rate is higher in men than women. In Taiwan, oral cancer (ICD9 code 140–149, excluding 142 and147) has shown the highest rate of increase among male cancers. The age-adjusted incidence rate has increased 11.3% from 1999 to 2008. In 2008, oral cancer was the fourth most common cancer in males with a sex ratio of 12.7∶1, male to female [1]. This male bias may be attributable to differences in prevalence of cigarette smoking, alcohol drinking and areca quid (AQ) chewing between the sexes [2]. About two thirds of oral cancers occur in the oral cavity. The primary treatment for oral cavity squamous cell carcinoma (OSCC) is radical surgery with or without post-operative chemoradiation [3]. In spite of improvements in the treatment of OSCC, the 5-year survival rate has remained almost unchanged over the past decade [4]. To date, the most important factors predicting outcome with OSCC are tumor volume, grade and TNM stage [5]. However, neither biological behavior nor response to therapy can be fully explained by these factors. If we can better understand the characteristics of OSCCs, this may ultimately help clinicians to provide OSCC patients with more appropriate treatment.

Studies on human cancers and animal models have indicated that tumor development proceeds via a succession of genetic changes in nuclear genome that lead to the progressive conversion of normal cells into cancer cells. Accordingly, Hanahan and Weinberg proposed that, among the vast catalog of cancer cell genotypes, six essential alterations in cell physiology were manifest; these lead to the genomic instability shared in common by all types of tumors [6]. In 2011, they added two new emerging hallmarks to the list, namely reprogramming of energy metabolism and evading of immune destruction [7]. Reprogramming of energy metabolism in cancer cells has been directly or indirectly linked to mitochondria and mitochondrial functional defects have been speculated to contribute to the development and progression of cancer [8].

In addition to furnishing cellular energy, mitochondria are involved in a wide range of signaling pathways associated with cell growth, differentiation and death [9]. A large volume of evidence has indicated that somatic mutations in mitochondrial DNA (mtDNA) are common in human cancers [10]. However, a comprehensive analysis of all reported mtDNA point mutations in human tumors has revealed that 72% are previously reported polymorphic variants [11]. Unique sets of mtDNA polymorphisms are able to define a human mtDNA haplogroup. These haplogroups are associated with region-specific mtDNA sequence variation that is the result of genetic drift and/or adaptive selection to environmentally favored mitochondrial functioning [12]. Difference in redox signaling as a consequence of haplogroup-associated oxidative phosphorylation capacity has been reported [13], [14], [15]. These functional differences may contribute to the susceptibility in relation to metabolic diseases, degenerative diseases, aging and cancer [16], [17].

It has been shown that mitochondrial haplogroups, and in some cases specific nonsynonymous single nucleotide polymorphisms (SNPs), are correlated with cancer development [18], [19], [20], [21], [22], [23], [24], [25]. Recent studies have further indicated that certain mtDNA SNPs may correlate with the prognosis of certain cancers, for example esophageal squamous cell carcinoma [26]. However, a comprehensive evaluation of common mtDNA haplogroups in relation to cancer clinical outcome has not been carried out as yet. In this study we report an association between geographic haplogroups and disease-free survival among 300 OSCC patients using whole genome sequencing on mtDNA and haplotyping by HaploGrep website program.

## Materials and Methods

### Patients and Sample Specimens

This study was approved by the Institutional Review Board of Chang Gung Memorial Hospital and undertaken according to the ethical guidelines of human investigation. Since the sex ratio (male versus female) of OSCC incidence in Taiwan was about 12.7∶1 after age adjustment, the present study consisted of 300 male patients diagnosed with primary OSCC (Table 1) who were admitted to Chang Gung Memorial Hospital, Lin-Kuo, during the period from March 1999 to October 2005. All cases were histologically confirmed and gave informed consent for participation before surgery. Information on their history, including cigarette smoking, alcohol drinking and areca quid (AQ) chewing, as well as general demographic information, was obtained by uniform interview by a well-trained technician using a questionnaire. For each case, the tumor and a corresponding adjacent normal sample were surgically dissected into small pieces, frozen immediately in liquid nitrogen and stored at –80°C. In addition, 10 ml of venous blood was drawn; this was separated into plasma, buffy coat cells and red blood cells by centrifugation within 18 h of obtaining the blood, and stored separately at –80°C. Genomic DNA for sequencing was purified from the patient’s buffy coat cells and tissue samples as previously described [27].

**Table 1:** Characteristics of the 300 OSCC patients.

Clinical parameters	Category	Results (%)
Age (year)		
	Mean±SD	51.6±11.3
	Range	27–82
Site of primary tumor [No. of patients (%)]		
	Tongue	104 (34.7)
	Bucca	105 (35.0)
	Others	91 (30.3)
Tumor stage [No. of patients (%)]		
	Stage I	52 (17.3)
	Stage II	75 (25.0)
	Stage III	38 (12.7)
	Stage IV	135 (45.0)
Lymph node metastasis [No. of patients (%)]		
	Negative	196 (65.3)
	Positive	104 (34.7)
	Extracapsular spread	
	Yes	72 (24.0)
	No	32 (10.7)
Differentiation [No. of patients (%)]		
	Well	116 (38.7)
	Moderate/Poor	184 (61.3)
Cigarette smoking [No. of patients (%)]		
	Yes	242 (80.7)
	No	58 (19.3)
Alcohol drinking [No. of patients (%)]		
	Yes	147 (49.0)
	No	153 (51.0)
Areca quid chewing [No. of patients (%)]		
	Yes	244 (81.3)
	No	56 (18.7)
Treatment regimen		
	Surgery only	124 (41.3)
	Surgery+Radiotherapy	121 (40.3)
	Surgery+Chemotherapy	2 (0.7)
	Surgery+Concomitant chemoradiotherapy (CCRT)	53 (17.7)

All of these 300 OSCC patients were undergone a wide excision of the primary tumors with 1 cm safety-margins (both peripheral and deep margins), which were cryosection checked. If a margin was positive for tumor involvement, an additional tissue was excised and checked to ensure that the margin was free from the tumor cells. All cases were histologically scored according to the recommendations for the reporting of specimens containing oral cavity, oropharynx and hypopharynx neoplasms by the Associations of Directors of Anatomical and Surgical Pathology (ADASP) [28]. Postoperative radiotherapy (RT) was routinely performed in patients who presented a stage pT4 tumor, pathologic positive lymph nodes, or pathologically close margins (≤4 mm). RT was scheduled within 4–8 weeks after surgery. The prescribed radiation dose was 1.8–2 Gy/fraction daily for 5 days per week. The total radiation dose was 66 Gy in patients who demonstrated multiple positive neck lymph nodes and/or extracapsular spread (ECS) and 60 Gy for the remaining patients. Concomitant chemoradiotherapy (CCRT) with cisplatin-based agents was also administered to patients with ECS or pathological multiple lymph node metastases [29]. All cases were followed up at the outpatient until death or until June 2011 according to the hospital guidelines of care. Briefly, all patients underwent a follow-up protocol of an outpatient visits every 1–6 months. The follow-up included physical examination, as well as hemogram, blood chemistry, chest X-ray and computed tomography (CT) scan or magnetic resonance imaging (MRI) when it was necessary. If patients who had abnormal clinical symptoms/signs or laboratory data, they further underwent a bone scan and liver ultrasound. The primary end-point was clinical recurrence, which was defined as relapse confirmed by histology or by an imaging study.

### PCR Direct Sequencing and Haplogroup Determination

Twenty-seven primer pairs (Table S1) covering the entire mtDNA genome, modified from a study by Wong et al. [30], were used for PCR amplification. Briefly, 100 ng genomic DNA was amplified by PCR for 30 cycles (30 s at 94°C, 30 s at 56–58°C, and 45–90 s at 72°C) in a final volume of 25 µL containing 1× PCR buffer, 1.5 mM MgCl_2_, 0.25 mM dNTP, 10 pmol primers, 1 U of *Taq* DNA polymerase (Geneaid) using a Mastercycler® gradient (Eppendorf AG, Hamburg, Germany). The DNA fragments were purified using a DNA PCR Clean-up kit (Geneaid) or by the ExoSAP (Exonuclease I - Shrimp Alkaline Phosphatase) method and subjected to direct sequencing. Both forward and reverse sequencing reactions were carried out using the same primers as the PCR amplification according to manufacturer’s instructions and analyzed on an ABI3130 Avent Genetic Analyzer (Applied Biosystems, Foster City, CA). Sequence variations were determined using ChromasPro v1.22 (Technelysium Pty Ltd, Australia) by comparing with a mtDNA reference sequence (NC_012920, revised Cambridge Reference Sequence (rCRS) of the human mitochondrial DNA). Conversion of the annotated mtDNA variants into accurate haplogroups was carried out by HaploGrep based on Phylotree build 14 (http://haplogrep.uibk.ac.at) [31]). An analysis of the quality of the haplogroup assignment showed that all of the cases was quite accurately/reliably grouped (quality score> = 80%, Table S2).

**Figure 1:**
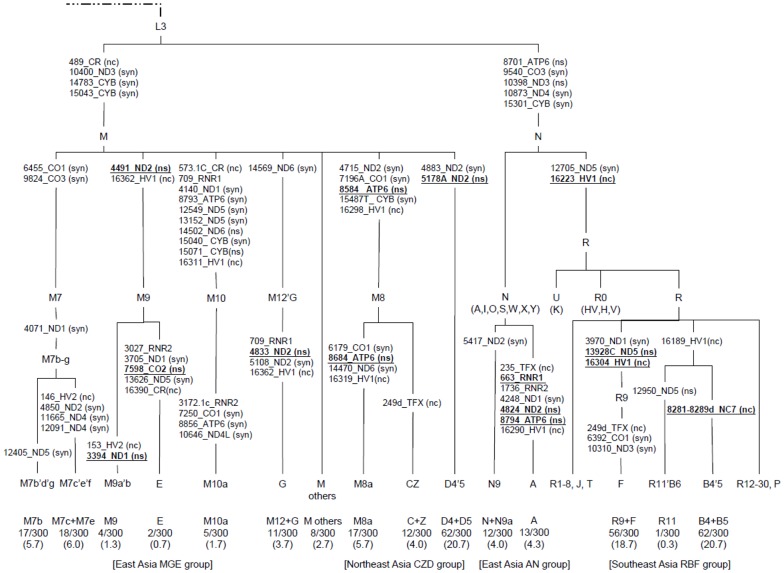
Classification tree of the mtDNA geographic haplogroups observed in 300 OSCC patients. The diagnostic variants are underlined.

### Prediction of Nonsynonymous Variant Functionality

A number of methods have been developed to predict the impact of base substitutions on protein structure and function. In the present study, five methods, namely the PolyPhen-2 algorithm, the SIFT (Sorting Intolerant From Tolerant) algorithm, the Grantham value, the BLOSUM 62 matrix, and the degree of evolutionary conservation, were applied to predict the putative effect of each nonsynonymous variant on protein function. The PolyPhen-2 algorithm (Polymorphism Phenotyping v2) predicts variants as “benign”, “possibly damaging”, or “probably damaging” using straightforward physical and comparative algorithms [32]. The SIFT algorithm predicts variants in the query sequence as “neutral” or “deleterious” using normalized probabilities calculated from the input sequence alignment [33]. The Grantham value (possible range from 5 to 215) is a measure of chemical similarity and value of <50 are classified as nonsynonymous conservative [34]. BLOSUM62 predicts how evolutionarily favorable a nonsynonymous variant is with scores range from +4 to −3, and variants with scores of <0 or of > = 0 are evolutionarily less or more favorable, respectively. Finally, the variants were classified as evolutionary conserved (EC) or non-conserved (EU) based on sequence alignments with ten mammalian orthologs using the mtSAP Evaluation from the GiiB-JST mtSNP (mitochondrial single nucleotide polymorphism) database [35].

### Statistical Analysis

All data were inputted into SPSS version 13.0 software in order to perform the statistical analysis and significant differences were assumed when *p*<0.05. Kaplan–Meier survival analysis was performed to observe the clustering of the geographic haplogroup curves. Univariate Cox regression survival analysis was used to test the survival distribution of haplogroups. A value *p*<0.05 was considered statistical significant. Multivariate Cox regression survival analysis was adjusted for age, cancer differentiation and cancer stage.

## Results

### Mitochondrial DNA Haplogroup and Geographic Haplogroup Classification

Based on HaploGrep, twenty haplogroups (A, B4, B5, C, D4, D5, E, F, G, M7b, M7c’e, M8a, M9, M10a, M12a, N9a, R9, R11, Z and M others) were found among our 300 OSCC cases (Table 2). All these 20 haplogroups were members of either the macro-haplogroup M or the macro-haplogroup N (Figure 1). The highest (20.7%) frequency among the Taiwanese OSCC patient mtDNA pool was observed for B4/5 followed by F (16.7%). In addition, moderately high frequencies for haplogroups D4 (12.0%) and D5 (8.7%) were found. According to phylogenetic analysis [36] and haplogroup geographic distribution, these 20 haplogroups can be clustered into four major geographic haplogroups, namely the East Asia AN (8.3%, 25/300), Southeast Asia RBF (39.7%, 119/300), East Asia MGE (21.7%, 65/300) and Northeast Asia CZD (30.3%, 91/300) groups.

**Table 2:** The distribution of mtDNA haplogroups in 300 Taiwanese OSCC patients.

Regional haplogroup	mtDNA Haplogroup	N (%)
East Asia MGE group		65 (21.7)
	M7b	17 (5.7)
	M7c’e	18 (6.0)
	M9	4 (1.3)
	M10a	5 (1.7)
	M12a	2 (0.7)
	M others	8 (2.7)
	G	9 (3.0)
	E	2 (0.7)
Northeast Asia CZD group		91 (30.3)
	M8a	17 (5.7)
	C	3 (1.0)
	Z	9 (3.0)
	D4	36 (12.0)
	D5	26 (8.7)
East Asia AN group		25 (8.3)
	A	13 (4.3)
	N/N9a	12 (4.0)
Southeast Asia RBF group		119 (39.7)
	R9	6 (2.0)
	R11	1 (0.3)
	B4/B5	62 (20.7)
	F	50 (16.7)

### Correlations of Geographic Haplogroups with Clinicopathological Parameters

**Figure 2:**
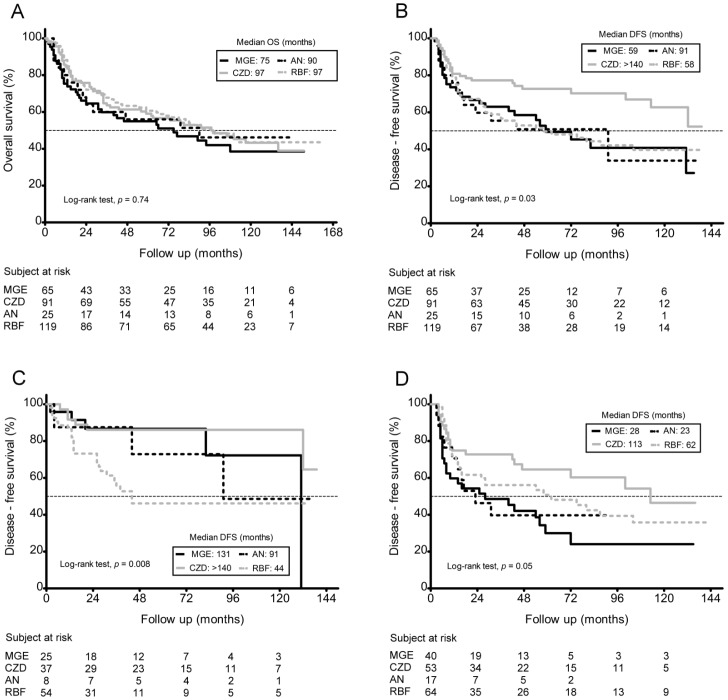
Kaplan–Meier survival curves for OSCC patients. A, overall survival (OS) based on mtDNA geographic haplogroups (*p* = 0.74). B, disease-free survival (DFS) according to mtDNA geographic haplogroups: Northeast Asia CZD haplogroup (n = 91), East Asia MGE haplogroup (n = 65), East Asia AN haplogroup (n = 25), and Southeast Asia RBF haplogroup (n = 119); *p* = 0.03. C, DFS according to mtDNA geographic haplogroups in patients treated with surgery alone (*p* = 0.008). D. DFS according to mtDNA geographic haplogroups in patients received radiotherapy after surgery (*p* = 0.05).

Geographic haplogroups were found not to be associated with any clinicopathological parameter including age, cigarette smoking, alcohol drinking, AQ chewing, tumor stage, differentiation and anatomic site (Table 3). On the other hand, the Kaplan-Meier plot analysis indicated that individuals who were members of the CZD haplogroup showed a significant association with better disease-free survival (DFS) than members of the other three geographic haplogroups (Figure 2B) and this phenomenon still existed after adjusting for tumor stage, differentiation and age at diagnosis (hazard ratio (HR) = 0.55; 95% confidence interval (CI) = 0.36–0.84) (Table 4). However, there was no difference in overall survival between the geographic haplogroups (Figure 2A).

**Table 3:** Associations between mtDNA geographic haplogroup and OSCC clinicopathological parameters.

Parameters	Category	East Asia MGE group N (%)	Northeast AsiaCZD group N (%)	East Asia AN group N (%)	Southeast Asia RBF group N (%)	*p* value
Total cases		65 (21.7)	91 (30.3)	25 (8.3)	119 (39.7)	
Age	<51 (n = 149)	37 (24.8)	40 (26.8)	12 (8.1)	60 (40.3)	0.46
	≥51 (n = 151)	28 (18.5)	51 (33.8)	13 (8.6)	59 (39.1)	
Cigarette smoking						0.18
	Yes (n = 242)	56 (23.1)	75 (31.0)	22 (9.1)	89 (36.8)	
	No (n = 58)	9 (15.5)	16 (27.6)	3 (5.2)	30 (51.7)	
Alcohol drinking						0.58
	Yes (n = 147)	36 (24.5)	43 (29.3)	10 (6.8)	58 (39.5)	
	No (n = 153)	29 (19.0)	48 (31.4)	15 (9.8)	61 (39.9)	
AQ chewing						0.52
	Yes (n = 244)	56 (23.0)	70 (28.7)	21 (8.6)	97 (39.8)	
	No (n = 56)	9 (16.1)	21 (37.5)	4 (7.1)	22 (39.3)	
Tumor stage						
	I/II (n = 127)	29 (22.8)	40 (31.5)	7 (5.5)	51 (40.2)	0.50
	III/IV (n = 173)	36 (20.8)	51 (29.5)	18 (10.4)	68 (39.3)	
Lymph node metastasis						0.97
	Negative (n = 196)	41 (20.9)	60 (30.6)	17 (8.7)	78 (39.8)	
	Positive (n = 104)	24 (23.1)	31 (29.8)	8 (7.7)	41 (39.4)	
	LNECS					
	Yes (n = 72)	18 (25.0)	21 (29.2)	5 (6.9)	28 (38.9)	
	No (n = 32)	6 (18.8)	10 (31.3)	3 (9.4)	13 (40.6)	
Differentiation						0.52
	Well (n = 116)	23 (19.8)	31 (26.7)	11 (9.5)	51 (44.0)	
	Moderate/poor (n = 184)	42 (22.8)	60 (32.6)	14 (7.6)	68 (37.0)	
Tumor site						
	Tongue (n = 104)	21 (20.2)	29 (27.9)	9 (8.7)	45 (43.3)	0.82
	Bucca (n = 105)	25 (23.8)	36 (34.3)	9 (8.6)	35 (33.3)	
	Others (n = 91)	19 (20.9)	26 (28.6)	7 (7.7)	39 (42.9)	

**Table 4:** Univariate and multivariate analysis of disease-free survival in 300 OSCC patients.

		Univariate		Multivariate	
Variables	Category	Hazard ratio (95% CI)	*p* value	Hazard ratio (95% CI)	*p* value
Age (years)					
	<51	1		1	
	≥51	0.84 (0.59–1.19)	0.32	1.0 (0.70–1.43)	0.99
Differentiation					
	Well	1		1	
	Moderate/poor	1.09 (0.76–1.56)	0.63	1.07 (0.74–1.54)	0.74
Primary tumor					
	T1/T2	1		1	
	T3/T4	1.48 (1.04–2.10)	0.03	1.24 (0.86–1.78)	0.26
Nodal status					
	(−) metastasis, (−) ECS	1		1	
	(+) metastasis, (−) ECS	1.39 (0.78–2.46)	0.26	1.28 (0.71–2.29)	0.41
	(+) metastasis, (+) ECS	2.39 (1.62–3.54)	<0.001	2.14 (1.41–3.25)	<0.001
mtDNA haplogroup					
	CZD haplogroup	0.52 (0.34–0.80)	0.003	0.55 (0.36–0.84)	0.006
	Others	1		1	

### Interactions between Geographic Haplogroups and Radiotherapy/chemo-radiotherapy with Respect to Disease-free Survival

A few studies have indicated that somatic mutations within tumor mtDNA may contribute to tumor growth [37], tumor cell metastasis [38] and chemoresistance [39]. Therefore, the effects of the haplogroups on prognosis with respect to different clinical regimes were evaluated. We found that individuals who were members of the CZD haplogroup had the best DFS among the four geographic haplogroups when treated after surgery either without RT/chemo-radiotherapy (CRT) or with RT/CRT (Figure 2C and 2D). In contrast, individuals within the RBF haplogroup showed a significant association with a poorer DFS than the other three haplogroups of patients when treated with surgery alone (Figure 2C). The median times to relapse were >140, 131, 91, and 44 months for the CZD, MGE, AN and RBF haplogroups, respectively. However, this was not the case for patients who had been treated with RT/CRT after surgery (Figure 2D). The median times to relapse were 113, 28, 23, and 62 months for the CZD, MGE, AN and RBF haplogroups, respectively, among patients who had been treated with RT/CRT after surgery.

## Discussion

The Taiwanese mtDNA branch of mitochondrial variants can be classified into four main geographic clusters (MGE, CZD, AN and RBF), which largely belong to three specific subhaplogroups of the Eurasian founder haplogroups, M, N and R (Figure 1). Haplogroup B and F, the oldest of the Han mtDNA haplogroups [40] were the top two haplogroups in the present study. The frequency of the other major mtDNA haplogroups (D, M7, M8, A) are also similar to previously published data [40], [41].

**Table 5:** Characteristics of specific nonsynonymous variants in four geographic haplogroups observed in 300 OSCC patients.

Haplogroup	Variant nucleotide position	Gene		Amino acid change	Respiratory complex involved	Prediction of variant functionality^a^						Frequency^b^(%)
						PolyPhen-2	SIFT^c^	Grantham	BLOSUM 62 matrix		Evolutionaryconservation^d^	
Macro-M haplogroup												
MGE/CZD	8701G	ATP6		T59A	V	Benign (0.00)	0.57 (T)	**58**		**−1**	EU	153/156 (98.1)
MGE/CZD	10398G	ND3		T114A	I	Benign (0.00)	0.36 (T)	**58**		**−1**	EU	156/156 (100)
East Asia MGE haplogroup (n = 65)												
M7b	4048A	ND1		D248N	I	Benign (0.00)	1.00 (T)	23		1	EU	16/17 (94.1)
M7b	5460A	ND2		A331T	I	Benign (0.00)	0.29 (T)	**58**		**−1**	EU	16/17 (94.1)
M7b	7853A	COII		V90I	IV	Benign (0.00)	1.00 (T)	29		3	EU	17/17 (100)
M7b	12811C	ND5		Y159H	I	Benign (0.00)	0.69 (T)	**83**		2	EU	14/17 (82.4)
M7c	5442C	ND2		F325L	I	Benign (0.00)	1.00 (T)	22		0	EU	16/18 (88.9)
M9 (M9a/E)	4491A	ND2		V8I	I	Benign (0.00)	1.00 (T)	29		3	EU	5/6 (83.3)
M9a	3394C	ND1		Y30H	I	Benign (0.01)	**0.04 (D)**	**83**		2	**EC**	4/5 (80.0)
E	7598A	COII		A5T	IV	Benign (0.00)	0.17 (T)	**58**		**−1**	EU	2/2 (100)
E	14577C	ND6		I33V	I	Benign (0.00)	0.28 (T)	29		3	**EC**	2/2 (100)
M12a	12030G	ND4		N424S	I	Benign (0.00)	0.38 (T)	46		1	EU	2/2 (100)
M12a	12358G	ND5		T8A	I	Unknown	0.48 (T)	**58**		**−1**	EU	2/2 (100)
M12a	15651T	Cyt b		A302V	III	Benign (0.14)	0.88 (T)	**64**		0	**EC**	2/2 (100)
G	4833G	ND2		T122A	I	**Possibly damaging (0.21)**	**0.02 (D)**	**58**		**−1**	EU	9/9 (100)
Northeast Asia CZD haplogroup (n = 91)												
M8 (M8a/CZ)	8584A		ATP6	A20T	V	Benign (0.06)	**0.03 (D)**	**58**		**−1**	EU	26/29 (89.7)
M8a	8684T		ATP6	T53I	V	Benign (0.00)	0.56 (T)	**89**		**−2**	EU	16/17 (94.1)
D (D4/D5)	5178A		ND2	L237M	I	**Possibly damaging (0.36)**	0.59 (T)	15		2	EU	61/62 (98.4)
D4	8414T		ATP8	L17F	V	**Probably damaging (0.99)**	0.30 (T)	22		0	EU	35/36 (97.2)
D5	5301G		ND2	I278V	I	Benign (0.00)	**0.01 (D)**	29		3	EU	25/25 (100)
East Asia AN haplogroup (n = 25)												
N/N9a	12358G		ND5	T8A	I	Unknown	0.48 (T)	**58**		**−1**	EU	9/12 (75.0)
A	4824G		ND2	T119A	I	**Possibly damaging (0.50)**	**0.05 (D)**	**58**		**−1**	**EC**	13/13 (100)
A	8794T		ATP6	H90Y	V	Benign (0.00)	0.10 (T)	**83**		2	**EC**	13/13 (100)
Southeast Asia RBF haplogroup (n = 119)												
R9/F	13928C		ND5	S531T	I	**Possibly damaging (0.21)**	1.00 (T)	46		1	EU	54/56 (96.4)

a Five methods including the PolyPhen-2 and SIFT (Sorting Intolerant From Tolerant) algorithm, the Grantham and BLOSUM 62 matrix, and the degree of evolutionary conservation wer applied to predict the putative effect of each nonsynonymous variant on protein function. Variant on protein function predicted to be significantly impacted is presented in bold phase.

b cases with variant/cases in the defined haplogroup.

c T: tolerated; D:deleterious.

d EC: evolutionary conserved; EU: evolutionary unconserved.

In the present study, a relationship between the mtDNA geographic haplogroups and DFS was observed in Taiwanese OSCC patients. The Northeast Asia CZD haplogroup was significantly associated with better DFS than the other three haplogroups after adjusting for tumor stage, differentiation and age at diagnosis (HR = 0.55; 95% CI = 0.36–0.84). Furthermore, an interaction between Southeast Asia RBF haplogroup and RT/CRT in DFS was noted. These findings indicated that membership of a particular mtDNA haplogroup among Taiwanese could affect the prognosis with respect to certain human cancer such as OSCCs.

It is generally accepted that mitochondrial reactive oxygen species (ROS) are likely to be important players in promoting tumor growth and metastasis [42], [43]. Recently, Taddei et al. [44] found that mitochondrial ROS produced by complex I defects of stromal fibroblasts are key molecules that are able to modulate the aggressiveness of surrounding cancer cells. In addition, common “non-pathological” mtDNA haplogroups have been found that determine differences in mitochondrial oxidative phosphorylation (OXPHOS) performance and ROS production both in mice and human [15], [45]. Recently, Gomez-Duran et al. [46] further provided the direct evidence that the mtDNA haplogroup differentially contributes to OXPHOS functionality using ‘cybrid’ model. Due to the difficulities in ‘cybrid’ analysis using archived clinical samples, a thorough phylogenetic analysis was carried out to ascertain whether unique variants existed in specific mtDNA haplogroups that might be associated with mitochondrial oxidative stress and thus might in part explain our findings. As shown in Table 5, the haplogroup variants mainly affect ATP synthase 6 (ATP6), NADH dehydrogenase subunit 1 (ND1), ND2, ND3, ND4, ND5, ND6, cytochrome b (Cyt b) and cytochrome c oxidase subunit 2 (COII); these mitochondrion-encoded proteins are the ones that are able to cause differences in OXPHOS coupling between the haplogroups [13], [14].

The Northeast Asia CZD haplogroup consists of haplogroup M8 (M8a+CZ) in Northeast Asia and haplogroup D (D4+D5) in Central/East Asia. Haplogroup M8 is characterized by the specific variant G8584A (A20T) and M8a contains an additional variant C8684T (T53I) in ATP6 gene. Protein secondary-structure modeling has indicated that A20T alters the hydrophobicity of the ATP6 protein and enhances the activity of the mitochondrial ATP synthase complex [47], while C8684T (T53I) is shown to result in a high physicochemical difference (Grantham value of 89) (Table 5). Since M8a has been shown to have a protective effect on clinical expression of Leber hereditary optic neuropathy (LHON) in Chinese families with the mtDNA G11778A mutation [40], it is possible that haplogroup M8 might also have a beneficial effect on DFS among Taiwanese OSCC patients. Haplogroup D is defined by the specific variation C5178A in the ND2 gene. Previous studies have demonstrated a protective effect of C5178A against oxidative damage to mitochondria [48], [49] and the accumulation of mtDNA mutations [50]. In addition, epidemiological studies have indicated that haplogroup D shows an association with longevity [51], [52] and also confers resistance against myocardial infarction [48], reduces the likelihood of onset of diabetes mellitus (DM) type 2 [53] and reduces the possibility of acute mountain sickness (AMS) [54]. Therefore, it seems possible that the protective effect of haplogroup D against oxidative damage might also be beneficial in terms of the OSCC clinical outcome, although, it has been reported that haplogroup D is associated with an increased risk of esophageal and thyroid cancer in China [55], [56].

The East Asia MGE haplogroup is comprised of the M7, M9, G and E haplogroups. It has been reported that the M7 haplogroup is associated with an increased risk of lung cancer [25] and AMS [54]. In addition, haplogroup M7b1’2, a sub-haplogroup of M7, has been found to be associated with an increase in the penetrance of LHON and a significant increase in the risk of visual loss [47]. Growing evidence indicates that ROS contribute to the pathophysiology of AMS [57]. However, the specific variation that defines the M7 haplogroup is a synonymous mutation T9824C in the cytochrome c oxidase subunit III gene. The relationships between haplogroup M7 and the ROS level are thus worthy of further investigation. Haplogroup M9 is defined by a specific variant at T3394C (Y30H) in an evolutionarily conserved region and may change the predicted secondary structure and the functioning of ND1 [58]. Haplogroup G has a specific variant at A4833G (T122A) in the ND2 gene that is predicted to have an effect on protein function (Table 5). Recently, Zheng et al. [25] reported that haplogroup G is associated with an increased risk of lung cancer and a higher frequency of mtDNA deletion in a Han Chinese population. Taken together, the East Asia MGE haplogroups consist of several key nonsynonymous variants in genes coding for mitochondrial respiratory-chain complexes I and III proteins that are known to be involved in ROS generation (Table 5). This might, at least in part, help to explain the unfavorable DFS found for these individuals in the present study.

Within the East Asia AN haplogroup, haplogroup A is defined by two specific variants, one at A663G in RNR1 (12S rRNA) gene and another at C8794T in the ATP6 gene. It has been reported that haplogroup A is associated with an increased risk of atherothrombotic cerebral infarction [59] and coronary atherosclerosis [60]. According to the core secondary structure model, the substitution A663G disrupts the Watson-Crick base pairing in the stem portion of the 1^st^ stem-loop structure of the 12S rRNA, which may result in altering its stability and thereby affecting its functioning [60]. The C8794T (H90Y) mutation of ATP6 is predicted to have an effect on protein function (Table 5), which may potentially alter the functioning of this enzyme. Furthermore, haplogroup A is also characterized by containing a variant at A4824G (T119A) in ND2 gene, which is predicted consistently to have an effect on this protein function (Table 5). Therefore, the association between haplogroup A and a poor DFS might be ascribable to contributions from these polymorphisms. It has been reported that sub-haplogroup N9 is negatively associated with longevity in a Rugao population [51]. On the other hand, haplogroup N9a, a sub-haplogroup of haplogroup N, has been reported to confer resistance against DM type 2 [61], and to protect against metabolic syndrome [62]. Among haplogroup N9a-specific nonsynonymous SNP, the A12358G (T8A) in the ND5 gene would seem to be essential for the function of complex I and might be one of the potentially functional polymorphisms. In this context, the underlying mechanisms associated with the polymorphisms within haplogroup N9 that are associated with a poor DFS are worthy of further investigation.

The Southeast Asia RBF haplogroup consists mainly of haplogroup B and F, two of the most common haplogroups in Han population [40]. Haplogroup F is defined by specific polymorphisms at nucleotides 16223 and 16304 in hypervariable region 1 (HV1) and G13928C (S531T) in the coding region of the ND5 gene, whereas haplogroup B is defined by two polymorphisms at nucleotides 16189 and 16223 in HV1, and its diagnostic marker is a 9-bp deletion in the COII/tRNA^Lys^ intergenic region (Figure 1). It has been reported that individuals with the 16223 variant showed a greater increase of VO_2max_ as a result of endurance training than those with wild type variant [63] and that the VO_2max_ is positively associated with ROS production [15]. Therefore, it is possible that the 16223 polymorphism might contribute to the unfavorable DFS found among OSCC cancer patients. Furthermore, the haplogroup F-specific nonsynonymous SNP, G13928C (S531T), is predicted to have an effect on ND5 protein function using PolyPhen-2 (Table 5). Komandur et al. [64] reported that the 9-bp deletion polymorphism might affect expression of downstream genes within the mtDNA and therefore alter ATP generation. Recently, it has been reported that haplogroup B is associated with an increased risk of developing severe AMS [54] and hepatocellular carcinoma [65], while sub-haplogroup B4a has been found to be negatively correlated with longevity [51]. Taken the above findings together, the poor DFS of OSCC patients who are members of the RBF haplogroup seem likely to be ascribable to contributions from their defined specific polymorphisms.

RT is commonly used in combination with surgery to treat OSCC patients who have advanced stage disease. Nonetheless, the DFS and OS of these OSCC patients with advanced stage disease are still poorer than those with early stage disease [5]. In the present study, we found that OSCC patients who received RT/CRT after surgery had an increased median DFS (62 months) compared to those with surgery only (44 months) among members of the RBF haplogroup. It is well-known that RT induces increased generation of ROS that exceed the protective capacity of the antioxidant mechanisms with in the cell and thus swamps the cell’s DNA repair system, which then leads to the death of the cancer cells. Hence, recent studies have shown that tumor cells with low levels of ROS contributed to radioresistance [66]. Thus, it is possible that haplogroup RBF might benefit from RT/CRT because these patients have a high baseline level of ROS as discussed above. Further studies are required to validate this hypothesis.

Recently, Hwang et al. [67] reported that nuclear gene expression is altered in response to possible differences in mitochondrial function that are related to different mtDNA haplogroups. This information suggests that the mtDNA haplogroups are able to define the cell’s genomic background and this is likely to play important role in tumor behavior. The results of the present study strongly support this idea and indicate that mtDNA haplogroups do affect the clinical outcomes of cancer patients. In addition, an interaction between haplogroup and RT/CRT in DFS was also noticed and this may have important implications for patients in terms of treatment choice.

## Supporting Information

Table S1 **Summary of primer sequences used to amplify the complete genome sequence of the human mitochondrion.**
(DOC)

Table S2 **Detailed information on the mtDNA genomic polymorphisms detected by direct sequencing and on the haplogroups as determined by HaploGrep (** http://haplogrep.uibk.ac.at **) for the 300 OSCC cases.**
(XLSX)
