# Supplementary material for: The Mitochondrial DNA Northeast Asia CZD Haplogroup Is Associated with Good Disease-Free Survival among Male Oral Squamous Cell Carcinoma Patients
Source: PLoS One. 2012 Nov 21;7(11):e49684. doi: 10.1371/journal.pone.0049684 (PMC3504154; doi:10.1371/journal.pone.0049684)
Supplement: Table S1 — Summary of primer sequences used to amplify the complete genome sequence of the human mitochondrion. (DOC) [file pone.0049684.s001.doc]

Table S1. Summary of primer sequences used to amplify the complete genome sequence of the human mitochondrion.

| **Amplified region** | **Forward primer** | **Reverse primer** | **Size, bp** |
| --- | --- | --- | --- |
| 12S | 5'-CAATGCAGCTCAAAACGCTT-3' | 5'-ATCCACCTTCGACCCTTAAG-3' | 655 |
| V | 5'- gcaagaaatgggctacattt -3' | 5'- TTCAATTTCTATCGCCTATACT -3' | 412 |
| 16s.1 | 5'-CCAGACAACCTTAGCCAAAC-3' | 5'-CCTTTCCTTATGAGCATGCC-3' | 749 |
| 16s.2 | 5'- GAACTGACAATTAACAGCCC -3' | 5'-TGTCCTTTCGTACAGGGAGG-3' | 775 |
| L-ND1-IQM | 5'-ATCCAGGTCGGTTTCTATCT-3' | 5'-GATGGTAGAGTAGATGACGG-3' | 1424 |
| ND2.1 | 5'-ATGTTGGTTATACCCTTCCC-3' | 5'-TTAGTGAGGGAGAGATTTGG-3' | 483 |
| ND2.2 | 5'- CCCATCTCAATCATATACCA -3' | 5'-TCTTGGTCTGTATTTAACCT-3' | 668 |
| WANCY | 5'-GCCCTTACCACGCTACTCCT-3' | 5'-CGAATAAGGAGGCTTAGAGC-3' | 557 |
| COI.1 | 5'-CCTATTATTCGGCGCATGAG-3' | 5'-GAAGCCTGGTAGGATAAGAA-3' | 697 |
| COI.2 | 5'-TTCTGATTTTTCGGTCACCC-3' | 5'-ATGAATGAGCCTACAGATGA-3' | 677 |
| SD | 5'-CCGATGCATACACCACATGA-3' | 5'-GTCGGTGTACTCGTAGGTTC-3' | 688 |
| K | 5'-AGTCCTCATCGCCCTCCCAT-3' | 5'- ATTTAGTTGGGGCATTTCAC -3' | 577 |
| ATP6 | 5'-CACTGTAAAGCTAACTTAGC-3' | 5'-TGAAAACGTAGGCTTGGATT-3' | 875 |
| COIII | 5'-TCACAATTCTAATTCTACTG-3' | 5'-CGGATGAAGCAGATAGTGAG-3' | 765 |
| GR1 | 5'-CGTCATTATTGGCTCAACTT-3' | 5'-TGGTAGGGGTAAAAGGAGGG-3' | 460 |
| GR2 | 5'-GACTACCACAACTCAACGGC-3' | 5'-GGGAGTGGGTGTTGAGGGTT-3' | 504 |
| ND4.1 | 5'- TCCTCCCTACTATGCCTAGA-3' | 5'-GCCAAGGTGGGGATAAGTGT-3' | 600 |
| ND4.2 | 5'- CAGCCACAGAACTAATCATA -3' | 5'-TTTGAGTTTGCTAGGCAGAA-3' | 667 |
| HSL | 5'-CCGGCGCAGTCATTCTCATA-3' | 5'- GGTTATAGTAGTGTGCATGG-3' | 673 |
| ND5.1 | 5'-CCCATGTCTAACAACATGGC-3' | 5'-GATTTGCCTGCTGCTGCTAG-3' | 769 |
| ND5.2 | 5'-AACGCTAATCCAAGCCTCAC-3' | 5'-TGAGAAATCCTGCGAATAGG-3' | 791 |
| ND6 | 5'-CATTAAACGCCTGGCAGCCG-3' | 5'-TATTTAGGGGGAATGATGGT-3' | 805 |
| E | 5'-GCTGTAGTATATCCAAAGAC-3' | 5'-TAATTACTGTGGCCCCTCAG-3' | 727 |
| Cytb-TP | 5'-GCAACAGCCTTCATAGGCTA-3' | 5'-GTACCGTACAATATTCATGG-3' | 1015 |
| Dloop1-2 | 5'-ATTACTGCCAGCCACCATGA-3' | 5'-GTTATTATTATGTCCTACAA-3' | 662 |
| Dloop2-3 | 5'- CGTGAAATCAATATCCCGCA -3' | 5'-GTGTGTGTGTGCTGGGTAGG-3' | 633 |
| Dloop3-F | 5'-TATCGCACCTACGTTCAATA-3' | 5'-GTTAATCACTGCTGTTTCCC-3' | 570 |
